# Current practice of colposuspension in the United Kingdom: Results of a national survey

**DOI:** 10.1111/aogs.70172

**Published:** 2026-03-11

**Authors:** Kar Yee Lor, Annika Taithongchai, George Araklitis, Aysha Waheed, Rayan Mohamed‐Ahmed, Angie Rantell, Dudley Robinson

**Affiliations:** ^1^ Kings College Hospital London UK; ^2^ Brunel University of London Uxbridge UK

**Keywords:** colposuspension, minimally invasive, preoperative, stress urinary incontinence, surgery, urodynamics

## Abstract

**Introduction:**

Colposuspension procedures have increased in the UK following the 2018 suspension of vaginal mesh for stress urinary incontinence. However, significant variation exists in preoperative assessment, patient selection, surgical approach, and postoperative care. This survey aims to explore current national practice.

**Material and Methods:**

A questionnaire was distributed to members of the British Society of Urogynaecology (BSUG) and the British Association of Urological Surgeons (BAUS).

**Results:**

Fifty‐three clinicians responded; most were gynecologists with a special interest in urogynecology (60%) or urogynecology subspecialists (34%). Response rates were 12% (51/442) among BSUG members and <1% (2/2622) among BAUS members. Annual case volume was 5 to 10 for 43%, fewer than 5 for 36%, 10–20 for 19%, and >20 for 2%. Most surgeons (85%) perform preoperative urodynamics in all patients, while 15% restrict testing to women with mixed symptoms. Almost all (98.1%) would treat detrusor overactivity or symptomatic overactive bladder prior to offering colposuspension. Forty‐five percent will perform colposuspension at any functional or maximum cytometric capacity; others use cutoffs of >400 mL (19%), >300 mL (26%) or >200 mL (10%). Fifty‐five percent reported a BMI limit of <35, while 19% have no limit. Most surgeons favored an open approach (58%), followed by laparoscopic (38%) and robotic (4%). Sutures were usually suspended tension‐free (75%) using Ethibond (53%) or PDS (40%), with 72% consenting for the use of non‐absorbable sutures. For open colposuspension, two sutures bilaterally were preferred (49%), followed by three (40%) and four (11%). Laparoscopic or robotic procedures were mostly intra‐peritoneal (89%), with surgeons placing two sutures bilaterally (88%) and tying their knots extracorporeally (75%). Thirty‐nine percent close the peritoneum over their sutures. Routine check cystoscopy was performed by 60%. Postoperatively, indwelling urinary catheters are mostly left on free drainage (69%); 17% always use a suprapubic catheter and 4% preferred clean intermittent self‐catheterization. Trial without catheter is most commonly attempted on Day 1 post‐colposuspension (74%), with variable voiding criteria and acceptable post‐void residuals.

**Conclusion:**

This national survey highlights marked heterogeneity in UK colposuspension practice. There is a need for comparative studies and national consensus on core technical steps to ensure safety and favorable long‐term outcomes.

## Graphical abstract

UK colposuspension practice is heterogenous. Comparative studies and national consensus on core technical steps are needed.

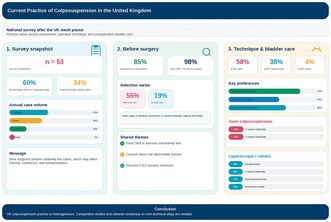

**Abbreviations**
BSUG: British Society of Urogynaecology
OAB: Overactive bladder
SUI: Stress urinary incontinence
UK: United Kingdom

**Key message**

UK Colposuspension practice varies widely across patient assessment and selection, operative technique, and postoperative care. Most clinicians treat overactive bladder symptoms/detrusor overactivity preoperatively and obtain consent for the use of non‐absorbable sutures. Comparative studies and national standards are needed to optimise patient outcomes.

## 1 INTRODUCTION

Stress urinary incontinence (SUI) is a prevalent condition which substantially affects the quality of life, psychosocial well‐being, and productivity of women worldwide.^1^, ^2^ For more than two decades, the synthetic mid‐urethral sling was the gold‐standard surgical treatment for SUI due to its high efficacy and minimally invasive nature.^3^ However, growing safety concerns and public scrutiny regarding mesh complications led to a pause on vaginally inserted mesh or tape for SUI in the UK.^4^ As a result, surgeons have had to revert to traditional continence surgeries—chief among them being colposuspension.^5^

Colposuspension is a well‐established SUI operation that predates mid‐urethral slings, with an overall cure rate of 68.9–88.0% for open colposuspension.^6^ Since its original description by John C. Burch, however, considerable variation has emerged in operative technique, including surgical approach, suture type, number of placements of sutures, and perioperative management.^7^ Introduction of laparoscopic and more recently robotic colposuspension allowed for a minimally invasive alternative.^8^ A systematic review comparing laparoscopic colposuspension with open colposuspension reported similar short‐term subjective cure rates, with the laparoscopic approach associated with a lower risk of perioperative complications.^9^

In 2020, bladder‐neck injections accounted for 64.5% of all SUI procedures in the UK, followed by colposuspension (22.4%) and autologous fascial slings (13.0%).^10^ In the absence of mesh‐based procedures, maintaining consistency of practice and ensuring sustainable training in colposuspension have become increasingly important. The objective of this national survey was to assess current colposuspension practices among UK gynecologists and urologists.

## 2 MATERIAL AND METHODS

A 25‐item Google Forms questionnaire (Table S1) was distributed to full members (consultant‐level) of the British Society of Urogynaecology (BSUG) and the British Association of Urological Surgeons (BAUS). The questionnaire was developed by the authors specifically for this study and focused on (i) preoperative patient selection, (ii) preoperative urodynamics parameters, (iii) operative techniques, and (iv) postoperative bladder care. Free‐text items were provided to capture description of respondents' trial without catheter protocol and management of overactive bladder symptoms prior to performing colposuspension.

The pilot version was tested by three urogynecology consultants as well as members of the BSUG research committee practicing in seven different hospitals across the UK, with revisions made based on their suggestions.

### 2.1 Data analysis

Quantitative and categorical data were analyzed using descriptive statistics in Microsoft Excel. Thematic analysis was performed for free‐text responses on the questionnaire.

## 3 RESULTS

A total of 53 responses were received: 60% were gynecologists with a special interest in urogynecology, 34% were urogynecology subspecialists, while 6% were urologists. The response rate was 12% (51/442) among full BSUG members and <1% (2/2622) of BAUS members.

Most respondents (88%) practice within National Health Service hospitals in England, with smaller proportions in Northern Ireland (6%) and Scotland (2%). Among those who work for the National Health Service, 71% work in district general hospitals, while 29% work in tertiary care hospitals. A small minority (4%) practiced exclusively in the private sector.

Almost half (43%) performed 5 to 10 colposuspensions per year; 36% performed less than 5, 19% performed 10–20 and only two percent reported more than 20 cases annually. Sixty‐nine percent of those who performed less than five colposuspensions per year practice at district general hospitals with a catchment population of between 270 000 and 600 000. Nearly all clinicians (93%) offer periurethral bulking agents, such as Bulkamid, while 81% also offer autologous fascial slings from either rectus sheath or fascia lata. Only one respondent, a urologist, offered implantation of an artificial urinary sphincter.

### 3.1 Preoperative patient selection and urodynamics

Most surgeons apply an upper BMI limit prior to offering colposuspension (81%). The most common threshold is <35 kg/m^2^ (55%), followed by <40 kg/m^2^ (15%) and <30 kg/m^2^ (11%). Ten surgeons (19%) reported no BMI cutoff.

**FIGURE 1:**
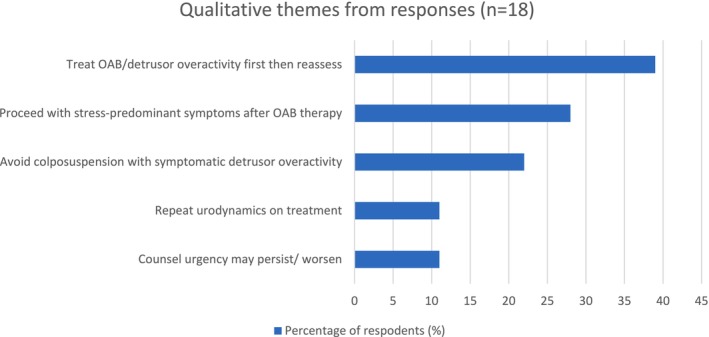
Qualitative themes from free‐text responses to Question 7‐ “Any comments regarding your management of detrusor overactivity or symptomatic OAB prior to offering colposuspension?”

Eighty‐five percent of clinicians would always perform preoperative urodynamics, while 15% reserve urodynamics only for patients who report mixed urinary symptoms. Almost all (98%) would treat symptomatic OAB or detrusor overactivity on urodynamics prior to offering a SUI procedure, such as colposuspension. Free‐text responses (*n* = 18) largely endorse a stepwise pathway: identify and treat OAB or detrusor overactivity first (sometimes with repeat urodynamics), and offer colposuspension only when SUI remains the dominant problem‐ with explicit counseling that urgency or detrusor overactivity may persist or worsen after colposuspension (Figure 1).

**FIGURE 2:**
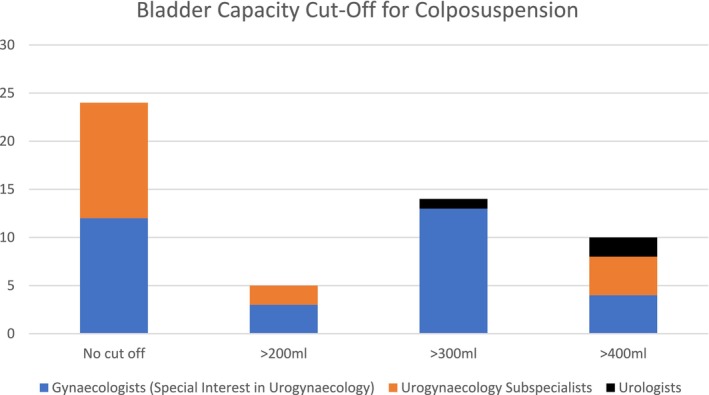
Responses to Question 5: “Do you have a cutoff for bladder capacity before considering colposuspension?”

Almost half (45%) will perform colposuspension at any functional or maximum cytometric capacity, while others have a cutoff at >400 mL (19%), >300 mL (26%) or >200 mL (10%). Variation in bladder‐capacity thresholds was comparable across the three specialties—gynecologists with a special interest in urogynecology, urogynecology subspecialists, and urologists (Figure 2).

Most surgeons (72%) will counsel and obtain patients' consent in the preoperative period for the placement of non‐absorbable sutures. One in five (21%) surgeons will also teach patients intermittent self‐catheterization in the preoperative period, particularly if there are features of voiding dysfunction on urodynamics.

### 3.2 Operative preferences and technique

Most surgeons still favor the open approach, with 58% performing predominantly open colposuspensions, while 38% performed mostly laparoscopic colposuspensions. Only 4% of surgeons performed the majority of their colposuspensions via the robotic approach.

Suture preferences varied: most respondents preferred Ethibond (53%), followed by PDS (polydioxanone; 40%). Fewer reported selecting Vicryl (polyglactin 910; 5%) or Monocryl (poliglecaprone 25; 2%).

Three out of four (75%) respondents suspend the vagina tension‐free from the ileo‐pectineal ligament, while the rest (25%) directly attach the vagina to the ileo‐pectineal ligament.

### 3.3 Open colposuspension

For open colposuspension, 49% preferred two suture placements bilaterally on the paravaginal fascia, followed by three (40%) and four bilaterally (11%).

### 3.4 Laparoscopic or robotic colposuspension

Almost all (89%) of laparoscopic or robotic procedures were performed intraperitoneally. Most laparoscopic or robotic surgeons preferred placing two sutures bilaterally 23/26 (88%); only 12% insert three sutures on each side of the paravaginal fascia. Three out of four (75%) minimally invasive surgeons tie their knots extracorporeally, while 25% tie their knots intracorporeally. Only 39% close the peritoneum over their sutures.

### 3.5 Cystoscopy

Sixty percent of surgeons will always perform a check cystoscopy post‐colposuspension, while 10% performed it only if there was a suspicion of a bladder injury. Among those who preferred using Ethibond sutures, routine postoperative cystoscopy was reported by 94% (16/17). Thirty percent of surgeons have never performed a check cystoscopy post‐colposuspension.

### 3.6 Bladder care

Following colposuspension, 17% of surgeons routinely insert a suprapubic catheter, while 15% do so selectively. Among those who leave an indwelling urinary catheter, most leave them on free drainage (69%), with 27% using flip‐flow. Only a minority (4%) of respondents prefer clean intermittent self‐catheterization initially.

**FIGURE 3:**
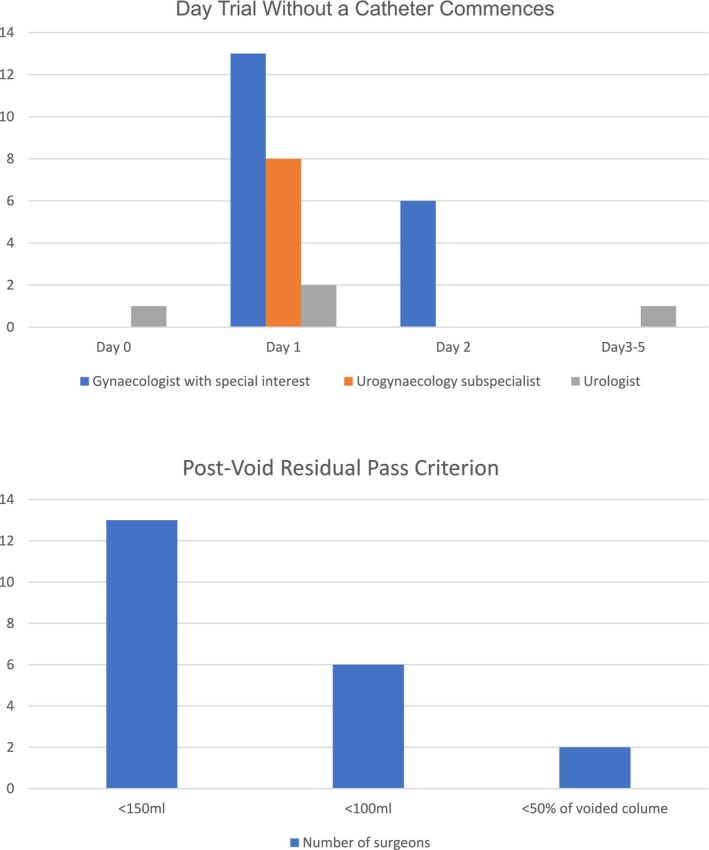
Responses to Question 23: “What is your trial without catheter protocol following colposuspension?”

There is a significant variation in clinicians' trial without catheter protocol (Figure 3). The majority (23/31, 74%) would remove patients' indwelling urinary catheters the next day (Day 1), while 20% remove them on Day 2. Interestingly, all urogynecology subspecialists' respondents (*n* = 8) unanimously preferred starting the trial without catheter on Day 1.

Number of voids during the trial without a catheter period ranged from 2 (11/15, 73%) to 3 (4/15, 27%). Among respondents who specified a pass criterion for post‐void residual, the most common threshold was a post‐void residual of <150 mL (13/21, 62%) followed by <100 mL (6/21, 29%). Fewer centers used a relative threshold of <50% of voided volume (9%).

## 4 DISCUSSION

This is the first national survey since 2003 assessing colposuspension practice in the UK, offering a timely snapshot on how surgeons have adapted following the suspension of mid‐urethral slings.^11^ The findings demonstrate that, beyond a few core principles, there is marked heterogeneity in techniques and preferences.

The variations in technique and preferences observed in the survey likely reflect diverse training backgrounds: some surgeons were trained in open Burch colposuspension during the pre‐mesh era, whereas others acquired surgical training more recently via short courses or mentorships during the mesh pause, often with an emphasis on laparoscopic or robotic approaches. By contrast, mid‐urethral slings are comparatively standardized and delivered via reproducible retropubic and transobturator approaches. They are also the most extensively studied surgical treatment for female SUI, supported by multiple randomized controlled trials, including head‐to‐head comparisons with colposuspension.^12^, ^13^, ^14^

This survey also highlights a generally low case load for colposuspension in the UK, with 79% of surgeons having fewer than 10 colposuspension cases per year. National audit data indicate that between 2020 and 2021, 321 laparoscopic colposuspensions, 357 open colposuspensions, and 394 autologous fascial slings were performed in the UK.^10^ By contrast, prior to the suspension of vaginally inserted mesh, substantially high volumes of mid‐urethral sling surgery were undertaken, with 4588 retropubic slings and 2060 transobturator tapes inserted in 2013 alone.^15^ The change in the medicolegal landscape in the UK meant that there is a requirement for only “appropriately skilled” surgeons to offer continence surgeries such as colposuspension. The reliance on mid‐urethral slings over the past two decades has also meant that training in colposuspension has declined accordingly, raising concerns about deskilling. Furthermore, the learning curve for colposuspension—particularly the laparoscopic and robotic approach—is steeper and longer than for mid‐urethral slings.^16^ This creates a self‐reinforcing barrier to competence and uptake. The low case numbers could also reflect an increasing surgical or patient preference toward bladder‐neck injections since the decline and pause in the use of mid‐urethral tapes.^15^

In the UK, guidelines from the National Institute for Health and Care Excellence (NG123) do not mandate preoperative urodynamics for women with pure SUI or stress‐predominant mixed urinary continence prior to SUI surgery. Despite this, the majority of respondents in this survey reported routinely performing preoperative urodynamics before offering colposuspension. These findings are consistent with data from a national BSUG audit of SUI surgery in the UK (2020–2021), which, under “high vigilance” requirements, reported urodynamic testing rates of 97–99% prior to colposuspension or autologous fascial sling procedures.^10^ Similar patterns were observed historically in the UK before the suspension of vaginally inserted mesh or tape for SUI, when preoperative urodynamics were undertaken in 90% of patients.^5^ However, it should be acknowledged that the BSUG database is based on voluntary, clinician‐entered data, and may not represent population‐level practice.

This apparent discrepancy between guideline recommendations and real‐world practice is reflected in broader European guidance. The European Urogynaecological Association working group concluded that while the evidence does not support the systematic use of preoperative urodynamics, urodynamics could be beneficial at anticipating postoperative outcomes in cases where mixed symptoms, voiding dysfunction, previous surgery, and concomitant prolapse are present.^17^ The recent medicolegal climate around incontinence surgery might also drive some to “cover all bases” by obtaining objective confirmation of urodynamic stress incontinence and bladder capacity, even if it infrequently changes surgical management.^18^ This variation underscores a need for a clear consensus on preoperative assessment specific to colposuspension.

There is also a lack of standardisation when it comes to patient selection, with a wide variation in BMI thresholds and in the management of concurrent OAB symptoms. A secondary analysis of 336 women suggests that BMI >25, age >60 years, and previous continence surgery are significant predictors of subjective surgical failure after open colposuspension.^19^ Another longitudinal study reported that preoperative weight >80 kg adversely affected both subjective and objective cure rates.^20^ Maximum functional or cystometric capacity criteria also varied in our cohort. In a risk‐factor analysis of 132 women, maximum cystometric capacity was not associated with postoperative urge incontinence 6 weeks after Burch colposuspension or autologous fascial sling (OR = 1.00; *p* = 0.14).^21^ There is a paucity of high‐quality studies assessing effect of bladder capacity on postoperative urinary urgency severity.

The observed heterogeneity in surgical technique likely reflects a field in transition. Before the widespread adoption of mid‐urethral slings, open Burch colposuspension was fairly standardized.^7^ Laparoscopic and robotic methods have introduced additional variability, and although short‐term outcomes appear comparable, robust data beyond 5 years remain scarce.^5^, ^9^ In our survey, 40% of surgeons reported placing three bilateral sutures at the paravaginal fascia during open procedures, compared with 12% during laparoscopic or robotic colposuspension. Although not assessed in our questionnaire, surgeons likely vary on how they determine “adequate” bladder‐neck elevation, introducing additional subjectivity.^22^ Such variation in suture number, type, placement, and intraoperative endpoints may influence durability and complications, yet there is a paucity of high‐quality comparative evidence. The national BSUG registry offers a valuable platform to check temporal trends in colposuspension outcomes and may help to inform future standardization efforts.

The study has several limitations. Firstly, the sample was modest, corresponding to a response rate of 12% from BSUG and <1% from BAUS. This raises the possibility of non‐response bias, which limits generalizability. Secondly, the survey targeted clinicians who currently perform colposuspension, and a low pool of providers may have further constrained responses and introduced selection bias. The survey also relied on self‐reported practices, which may be subject to response bias. Finally, although the questionnaire underwent piloting by three urogynecology consultants and members of the BSUG research committee, it was not formally validated.

## 5 CONCLUSION

This national survey demonstrates marked heterogeneity in current colposuspension practice in the UK, encompassing patient selection, perioperative assessment, operative technique, and postoperative bladder care. There is, however, clear consensus on treating overactive bladder or detrusor overactivity preoperatively and explicitly consenting patients for the use of non‐absorbable sutures. The generally low case volumes observed highlight ongoing challenges in maintaining surgical expertise and sustainable training in the post‐mesh era, supporting consideration of greater centralization within high‐volume units with appropriate expertise and infrastructure.

There is an urgent need for consensus on core technical steps and standardised perioperative protocols to support training and to ensure both patient safety and long‐term outcomes. Addressing these gaps will require prospective, multicenter comparative studies with robust long‐term outcome measurements.

Finally, comprehensive national data collection will be essential to underpin these efforts. While previous UK databases relied on voluntary clinician‐reported data, the recently introduced, nationally mandated Pelvic Organ Prolapse and Stress Urinary Incontinence (POPSUI) registry offers an important opportunity to systemically capture practice patterns, complications, and patient‐reported outcomes to inform future standardization and evidence‐based guideline development.

## AUTHOR CONTRIBUTIONS

Kar Yee Lor: survey distribution, data collection, and manuscript writing. Annika Taithongchai: survey design and editing, and manuscript editing. Aysha Waheed, Rayan Mohamed‐Ahmed, George Araklitis, Angie Rantell, and Dudley Robinson: manuscript editing.

## CONFLICT OF INTEREST STATEMENT

Mr. D Robinson is the Vice President of the International Urogynecological Association.

## ETHICS STATEMENT

Formal ethics approval was not required as the study surveyed clinicians anonymously, and patient‐identifiable data were not collected.

## Supporting information


Table S1.

## Data Availability

Data sharing is not applicable to this article as no datasets were generated or analyzed during the current study.
