# Supplementary material for: Current practice of colposuspension in the United Kingdom: Results of a national survey
Source: Acta Obstet Gynecol Scand. 2026 Mar 11;105(7):1398–404. doi: 10.1111/aogs.70172 (PMC13308968; doi:10.1111/aogs.70172)
Supplement: Supplementary file 1 — Table S1. [file AOGS-105-1398-s001.docx]

Table 1: Questions included in the 25-item survey along with question type and response options.

Introduction:

We are conducting a survey on current colposuspension practices across the United Kingdom. With the increase in colposuspension procedures following the pause on vaginal mesh, we aim to understand how surgical practices vary between different units.

| Question | Question type | Response Options |
| --- | --- | --- |
| Which hospital(s) do you work in | Free text option |  |
| Are you… | Single choice | A urologist  A urogynaecology subspecialist  A gynaecologist with special interest in urogynaecology |
| Are you offering other stress incontinence procedures in addition to colposuspension? | Multiple choice | No  Fascial sling (rectus or fascia lata)  Bulkamid or periurethral bulking agents  Artificial urinary sphincter |
| Do you perform pre-operative urodynamics? | Single choice | Yes-always  Sometimes depending if pure SUI or mixed symptoms  Never |
| Do you have a cut off for bladder capacity before considering colposuspension? | Single choice | Will perform on any bladder capacity  >400ml cut off  >300ml cut off  >200 cut off |
| If detrusor overactivity is present on urodynamics and the patient is symptomatic of overactive bladder, would you treat this prior to offering a SUI procedure? | Single choice | Yes  No |
| Any comments regarding your management of detrusor overactivity or symptomatic OAB prior to offering colposuspension? | Free text option |  |
| Do you have a BMI cut-off for offering colposuspension? | Single choice | <40  <35  <30  No |
| How many colposuspension procedures do you perform per year? | Single choice | 0-5  5-10  10-20  >20 |
| Are the majority of your colposuspensions performed | Single choice | Open  Laparoscopic  Robotic |
| If minimally invasive, do you perform the procedure intra-peritoneally or extra-peritoneally | Single choice | Intra-peritoneally  Extra-peritoneally |
| How many sutures do you place on either side if performing open colposuspension? | Single choice | 1  2  3  4  Not applicable |
| How many sutures for you place on either side if performing the procedure laparoscopically or robotically | Single choice | 1  2  3  4  Not applicable |
| Which suture material do you use | Multiple choice | PDS (polydioxanone)  Monocryl  Ethibond  Vicryl  V-loc  Other |
| If other, please specify | Free text option |  |
| If using a non-absorbable suture, do you consent the patient for permanent suture placement? | Single choice | Yes  No |
| If performing the procedure laparoscopically, how you tie your knots? | Single choice | Extracorporeal  Intracorporeal |
| Do you… | Single choice | Suspend the vagina from the ileo-pectineal ligament (tension free)  Directly attach the vagina to the ileo-pectineal ligament (traditional approach) |
| Do you close the peritoneum over your sutures | Single choice | Yes  No |
| Do you perform a check cystoscopy post-colposuspension? | Single choice | No  Yes- always  Yes- sometimes |
| If you’ve answered Yes- sometimes, please elaborate | Free text option |  |
| Do you also insert a suprapubic catheter | Single choice | Always  Never  On occasion |
| What is your trial without catheter (TWOC) protocol following colposuspension? | Free text option |  |
| Do you teach your patients clean intermittent self-catheterisation prior to proceeding with the procedure? | Single choice | Yes  No |
| If you leave a urinary catheter in place, do you… | Single choice | Leave it on free drainage  Leave it on flip-flow  I prefer clean intermittent self-catheterisation |
| Any other comments? | Free text option |  |
